# Effect of social influence, environmental awareness, and safety affordance on actual use of 5G technologies among Chinese students

**DOI:** 10.1038/s41598-023-50078-4

**Published:** 2023-12-17

**Authors:** Muhammad Farrukh Shahzad, Shuo Xu, Kanwal Iqbal Khan, Muhammad Faisal Hasnain

**Affiliations:** 1 College of Economics and Management, Beijing University of Technology, Beijing, 100124 People’s Republic of China; 2 Department of Management Sciences, University of Engineering and Technology, New Campus, Kala Shah Kaku, Pakistan; 3 Department of Chemistry, University of Agriculture, Faisalabad, 38000 Pakistan

**Keywords:** Environmental sciences, Environmental social sciences, Health care

## Abstract

5G technology continues to evolve, and its potential to revolutionize various aspects of society is becoming increasingly evident. However, the successful adoption and utilization of 5G technology depends on various factors, particularly among consumers expected to be early adopters and critical drivers of technological advancements. This study investigates the effect of social influence, environmental awareness, and safety affordance on Chinese university students' actual use of 5G (AU5G) technologies. It also analyzed the mediating role of trustworthiness and intention to use 5G (ITU5G) between them and the moderating role of facilitation conditions between trustworthiness and ITU5G. Data was collected from university students (n = 364) in Beijing and was examined employing the structural equation modelling (SEM) approach. The outcomes confirm that trustworthiness and ITU5G significantly mediate the relationship between social influence, environmental awareness, and safety affordance on AU5G technologies among Chinese students. Further, the supportive facilitation conditions strengthen the relationship between trustworthiness and ITU5G. These findings are backed by UTAUT2 models that support the technology acceptance and adoption among the users. The study outcomes can help policymakers design strategies to address potential barriers and encourage greater uptake of 5G services among university students.

## Introduction

Considering the information technology background and introducing technological advancement through fifth-generation (5G) is expected to expand the mobile communication sector further^1^. The data usage for 5G was anticipated to increase between 1.7 to 2.7 times that of 4G in six central South Korea, Japan, Germany, USA, UK, and Australia^2^. 5G is anticipated to deliver new business improvements and opportunities due to its innovative features and technological standards. It reshaped how individuals communicate, access information, and interact with the world^3^. In recent years, the emergence of 5G wireless technology has garnered considerable attention due to its potential to revolutionize connectivity and enable a wide range of innovative applications^4^. Being at the forefront of technological advancements, China has made significant strides in deploying and adopting 5G technology. However, as with any technological transition, the intention to use 5G (ITU5G) among different user groups, particularly university students, is influenced by various factors^5^. China's rapid economic growth and extensive digital infrastructure have laid a solid foundation for adopting 5G technology.

The Chinese government has firmly committed to developing and implementing 5G technology nationwide. This commitment is reflected in China's aggressive deployment of 5G infrastructure, making it one of the largest and fastest-growing 5G markets globally^6^. The Chinese government has made substantial advancements in telecommunications and has several leading technology companies driving the development and deployment of 5G networks and devices^7^. Huawei, ZTE, and other Chinese companies have been instrumental in developing 5G technologies, contributing to their adoption in the country. Consumers in China have demonstrated an excellent desire for cutting-edge technology and mobile connectivity. Many people want quicker internet, better data services, and better user experiences. Telecommunications companies have been encouraged by this consumer demand to invest in 5G networks to fulfill their customers' rising expectations^8^.

Universities can play a crucial role in disseminating accurate information to raise awareness and understanding among students. 5G technology helps in creating ease for the student's learning. It provides quick access to reliable information sources. When students believe in the benefits, applications, and potential of 5G, they are more likely to adopt it. Peer influence, recommendations from friends, and social media significantly shape individuals' attitudes and attitudes toward technology adoption^7^. In China, where social connections and networks hold immense importance^9^, the opinions and experiences shared by peers can significantly impact an individual's decision to adopt 5G technology. Positive word-of-mouth, social endorsements, and influencers promoting the benefits of 5G can create a sense of social norm and encourage students to embrace technology^10^. Students' awareness of the environmental impact of technology can influence their decision to adopt 5G. If they perceive 5G technology as environmentally friendly or as having the potential to support sustainable practices, it may encourage them to embrace it^11^. Nowadays, safety is a critical concern when adopting any new technology. If students perceive 5G technology as safe, with robust security measures and safeguards in place, they are more likely to embrace it. Universities and technology providers should prioritize addressing safety concerns and providing evidence of the security measures implemented in 5G infrastructure^12^.

Trust plays a vital role in adopting 5G technology and has the potential for transformative applications and services. Students may be more inclined to adopt 5G technology if they trust the institutions, organizations, and companies involved in its deployment and operation^13^. 5G technology enables the Internet of Things (IoT), augmented reality (AR), virtual reality (VR), and other innovative applications that have the potential to reshape various sectors, including education, healthcare, entertainment, and transportation^14^. Accessing advanced educational tools, immersive learning experiences, and remote collaboration opportunities through 5G networks can be highly appealing for university students. The promise of new and exciting possibilities through 5G technology motivates students to adopt and explore its potential benefits^15^. The support and regulatory framework provided by the government can significantly influence the adoption of 5G technology. The Chinese government actively promotes and supports the deployment of 5G infrastructure, including providing incentives, favorable policies, and regulatory frameworks; it can create an environment conducive to adoption among university students^5^.

One key factor driving the adoption of 5G technology among university students is the increased demand for faster and more reliable connectivity^16^. With the proliferation of smart devices and data-intensive applications, university students rely heavily on Internet connectivity for online learning, research, social networking, and entertainment^17^. The enhanced network capabilities of 5G technology offer the potential to deliver significantly faster download and upload speeds, reduced latency, and increased network capacity. These benefits can substantially enhance the user experience and enable seamless access to resource-intensive applications, thereby meeting the growing demands of tech-savvy university students^7^.

Prior research has examined the factors influencing consumers' intentional and actual usage decisions to adopt technology^18–20^. Only a few studies examined the factors influencing consumers' intentions to adopt 5G technology^7,21^, while very few researchers also discuss the actual use of 5G (AU5G) and provide theoretical explanation relating to the goal and application of existing technology^22,23^. Although these ideas offer a perception of the technological factors that encourage technology adoption, they only provide a limited understanding that supports the usage of new 5G technology. Considering the risk associated with 5G technology, safety, trust, and environmental factors will influence future consumers' AU5G. This study examines customers' attitudes toward 5G technology to understand the factors influencing acceptability and usage. This study addresses the two research questions. First, do trustworthiness and ITU5G significantly mediate the relationship between social influence, environmental awareness, and safety affordance on Chinese university students' AU5G technologies? Second, do facilitation conditions significantly moderate the relationship between trustworthiness and ITU5G?

This comprehensive analysis significantly contributed to the literature on technology adoption and UTAUT2 models. First, it advances adoption research by providing a theoretical model to analyze factors impacting user acceptability of 5G technology products and services. Second, based on the methodology, this research aims to explore the factors such as social influence, environmental awareness, safety affordance, trustworthiness, and ITU5G that affect the adoption of 5G technology among university students in China. This will fill the gap suggested by^5,24^. Third, it uses the structural equation modelling (SEM) method to examine the links' empirical robustness in the established framework. The unified theory of acceptance and use of technology (UTAUT2) posits that individuals are more likely to adopt a technology if they perceive it as useful and easy to use^12,23^. For university students, the perceived usefulness of 5G technology could be evaluated based on how well it aligns with their academic and personal needs. Students perceive 5G as a valuable tool for enhancing their educational experience, facilitating research, or supporting their daily activities, and they are likelier to adopt it^23^. In this way, it will theoretically make a contribution.

## Literature review with hypothesis development

### Unified theory of acceptance and use of technology (UTAUT2)

After incorporating innovative components into the unified theory of acceptance and use of technology (UTAUT2), we proposed a novel model to explain better how people use the internet and what factors affect their choice to use 5G technology^25^. The goal of^26^, is to evaluate and contrast several current theories, develop an adoption approach, and empirically support it. The upgraded version of UTAUT, introduced in 2003 by^26^, is called UTAUT2. It is one of the most well-known and extensively used models for technology adoption. It is the most relevant model for our study because it used to adopt mobile internet in 2012. Its additional significant characteristics can be found in further studies, enabling UTAUT2 to be employed in a wider variety of consumer technology adoption. The goal and behavioral expansions of the UTAUT2 model require more investigation^5^. The major measurement models of the study, such as social influence, environmental awareness, safety affordance, trustworthiness, facilitation conditions, ITU5G and AU5G, were validated. Our study used two parameters of the UTAUT2 theory, such as social influence and facilitation conditions, to support our conceptual framework.

In addition, UTAUT2 requested internet access to send SMS and MMS, download ringtones and logos, play Java games, use mobile email, and other things^12^. Utilizing mobile internet while maintaining the speed and quality of internet services was a cutting-edge feature at the time. Today, mobile internet is used for much more than just this, including social media browsing, online shopping, e-payments, navigation, attending conferences, and presenting work^27^. In summary, we can say that internet usage, as well as the perceived and actual advantages and uses of it, are significantly more complex than they have ever been. Close relationships and a creative attitude frequently influence the effectiveness and simplicity of a tool^28^. We think individual traits, including economic and social aspects, impact how people perceive and absorb innovations. According to^7^, it is crucial to consider particular psychological elements influencing decision-making while considering technology adoption. For a new technology marketing campaign to be successful, researchers advised including these elements^12,29^. We, therefore, created a model customized for the requirements of 5G internet technology to aid in the present research. Finding the antecedent thoughts from the past study^30^ and individual intentions and goals is prioritized and supports our model.

### Mediating role of trustworthiness and ITU5G

University students are essential to consider when investigating the use of 5G, as they are tech-savvy and early adopters of new technologies. The 5G technology encompasses understanding the technical aspects, benefits, and potential impact on the education industry^31^. Understanding the features and advantages of 5G, such as faster download and upload speeds, lower latency, and greater network capacity, can generate positive attitudes toward its usage^32^. Additionally, being informed about the potential applications of 5G in areas like healthcare, autonomous vehicles, smart cities, and the Internet of Things (IoT) can spark interest and curiosity among students^27^. Students with comprehensive knowledge of 5G are more likely to recognize its potential to improve their academic and research activities, enhance communication and collaboration, and provide opportunities for innovative applications in their fields of study^33^. Information acquisition helps address concerns and skepticism surrounding 5G technology. The spread of misinformation and misconceptions about potential health risks, privacy issues, and security vulnerabilities associated with 5G can create doubts and hesitations. However, when students actively seek accurate and reliable information from credible sources, they can make informed decisions and alleviate their concerns^34^, increasing their ITU5G and leading to AU5G.

The social influence of 5G technologies positively impacted the AU5G. By knowing about 5G, students can disseminate accurate information to their peers, promoting positive perceptions and intentions to use the technology^10^. This influence can further enhance the adoption of 5G among university students in China. Universities and educational institutions can organize workshops, seminars, and courses to educate students about 5G. These initiatives can cover the technical aspects of 5G, its applications across various domains, and its potential impact on future career prospects. Inviting industry experts and practitioners to share their insights can give students valuable knowledge and real-world examples^7^. Collaborative efforts between telecommunications companies, government agencies, and educational institutions can be instrumental in raising awareness about 5G. These campaigns can utilize various channels, such as social media, campus events, and informative websites, to disseminate accurate information and address common misconceptions^35^. Developing engaging and interactive content, such as videos, infographics, and case studies, can enhance students' interest in acquiring information about 5G. When individuals gather relevant information about 5G, its benefits, and potential applications, they can make more informed decisions about adopting and utilizing the technology^36^.

People's propensity to adopt and use new technologies, particularly 5G, is greatly influenced by their trust in the technology. When individuals perceive a technology or its providers as trustworthy, they are more likely to embrace and utilize it^12^. Trustworthy 5G providers establish a reputation for reliability and consistent service quality. Users believe that 5G will regularly supply fast and dependable connectivity and are more likely to accept and use it^37^. A past study^38^ highlighted trustworthy 5G networks prioritize security measures to protect user data and privacy. When people trust that their personal information is secure on the 5G network, they are more willing to engage with the technology. Transparent communication from 5G providers about network capabilities, coverage, and potential limitations builds trust among users^18^. When users clearly understand what to expect from 5G, they are more likely to develop positive intentions toward its use.

Trustworthy 5G providers adhere to ethical practices and demonstrate responsible behaviour. This includes addressing potential health concerns, respecting user rights, and ensuring equitable access to 5G services^39^. When users trust that the technology is developed and deployed responsibly, their intention to use it increases. Trustworthy 5G providers prioritize user experience and actively seek feedback to improve their services^7^. By valuing user satisfaction and continuously enhancing the 5G experience, providers can cultivate trust among users, leading to a higher intention to use the technology. Trustworthiness significantly shapes users' intentions to adopt and utilize 5G technology^40^. Individuals observe 5G technology and its providers as trustworthy; they are more likely to develop positive attitudes and intentions toward its use. Trustworthiness is closely associated with the reliability of 5G technology and its providers^28^. Users believe that 5G technology is reliable and consistently delivers the promised benefits; they are likelier to trust and develop an intention to use it.

Trustworthiness encompasses aspects such as network stability, signal strength, and consistent high-speed connectivity, which are crucial factors in adopting 5G^41^. Trust in 5G technology also relates to security and privacy concerns. Users are more inclined to use 5G because they trust that their personal information and data will be adequately protected. Assurance of secure connections and data encryption can enhance trustworthiness, increasing people's intention to adopt technology^42^. Open and transparent communication from 5G providers can contribute to trustworthiness. The experiences and opinions of others can also influence trust. Users observe their peers or authoritative figures using 5G technology, and having positive experiences can enhance their trust and ITU5G^43^. Trustworthy 5G providers offer reliable customer support and service. Past studies^7,18^ highlighted individuals believe that their concerns will be promptly addressed, which enhances their trust in the 5G technology. Supportive services, such as technical assistance, troubleshooting, and responsiveness to user feedback, contribute to a positive perception of trustworthiness^14^. Reliability, security, transparency, positive social influence, and quality service are all factors that contribute to trust in 5G technology and its providers^44^. Building and maintaining trustworthiness can help promote the adoption and successful implementation of 5G among university students^45^. Hence, we hypothesize that,

#### Hypothesis 1

Trustworthiness and ITU5G significantly mediate the relationship between social influence and AU5G.

As society becomes more aware of environmental issues and the need for sustainable practices, individuals are increasingly considering the environmental implications of their choices, including adopting new technologies like 5G^46^. Environmental awareness prompts individuals to seek information about what is going on in their surroundings. Concerns about climate change and energy consumption may lead people to prioritize technologies with a lower environmental impact. Regarding 5G, users may be interested in understanding the energy efficiency of the infrastructure and devices associated with the technology^21^. If they perceive 5G to be more energy-efficient than previous technologies, it may positively influence their intention to use it. The deployment of 5G networks involves the installation of additional infrastructure, such as small cell towers and base stations. Environmental awareness may drive individuals to consider the ecological impact of this infrastructure^47^. They may support 5G deployment plans that prioritize minimizing the environmental footprint, such as using renewable energy sources, considering wildlife habitats, and employing responsible waste management practices.

The proliferation of new technologies often increases electronic waste (e-waste) as older devices are replaced. Environmental awareness can make individuals more conscious of the e-waste generated by upgrading to 5G-compatible devices. They may consider factors like device durability, repairability, and recyclability before embracing 5G technology^48^. Environmental awareness can also influence the perception of the benefits that 5G can bring to sustainability efforts. It may create a trustworthy relationship between the technology provider and the user. For instance, 5G enables advancements in various sectors, such as smart cities, transportation, and agriculture, which can reduce greenhouse gas emissions, optimize resource utilization, and enhance environmental monitoring^49^. Highlighting these green applications can positively impact the intention to use 5G among environmentally conscious individuals. It creates trustworthy relationships between parties that ultimately increase the AU5G technology.

Ecologically aware students may consider the environmental impact of 5G technology. They may be concerned about the energy consumption and carbon emissions associated with the infrastructure required to support 5G networks^50^. Environmental awareness can make students more conscious of electronic waste and the ecological consequences of increased electronic consumption. Some environmentally conscious students may prioritize using eco-friendly alternatives over adopting new technologies. Environmental awareness played a significant role in telling the advantages of this new technology, such as speed and easy availability^51^. Although evidence from past studies^37^ suggested that 5G is safe, concerns about electromagnetic radiation and its impact on human health can influence students' intentions to use 5G. Environmental campaigns and awareness-raising initiatives can shape public opinion and influence attitudes toward 5G technology^24^. In past studies, it is essential to note that environmental awareness led to positive ITU5G, which may lead towards AU5G. Transparent communication and access to reliable research on these topics can help alleviate concerns and foster trust in the 5G technology^52^, which enhances ITU5G and positively increases the AU5G technologies among Chinese students. Hence, we hypothesize that,

#### Hypothesis 2

Trustworthiness and ITU5G significantly mediate the relationship between environmental awareness and AU5G.

Safety affordance refers to the perceived level of safety and security provided by a particular technology^7^. Regarding 5G technology, the perception of safety affordance can significantly impact users' intention to adopt and use it^36^. One of the primary factors influencing people's perception of safety affordance in 5G technology is the concern about potential health risks^13^. Some individuals may worry about the increased electromagnetic radiation associated with 5G networks and its impact on human health. People perceive 5G technology as a potential threat to their well-being; they may be less inclined to use it^53^. That is why trustworthiness is an important element built when users believe the company adopts sufficient safety measures before launching a new technology. It is supported by a previous study^54^ that 5G technology has no harmful effect on health. Due to 5G's ability to facilitate the proliferation of the Internet of Things (IoT) devices and connected infrastructure, safety affordance also extends to data security and privacy concerns, which increases trustworthiness.

Users of 5G technology know about security measures, so they are willing to adopt the technology without fearing data breaches or unauthorized access to their personal information. Safety affordance in the context of 5G also encompasses network reliability and resilience. Users want a seamless and uninterrupted experience when using 5G services^55^. Users' trust also influences the perceived safety affordance of 5G technology in the technology providers and network operators. To enhance the ITU5G technology, stakeholders such as telecommunication companies, regulatory bodies, and governments must address these safety affordance concerns^56^. Safety affordance is achieved through transparent communication about the technology's safety measures, addressing health concerns with scientific evidence, implementing security protocols, ensuring network reliability, and fostering trust through responsible practices^57^.

Safety affordance refers to the perception of safety features or measures in a technological system that influences users' ITU5G technology. Safety affordance in 5G technology enhances trust and reliability among users. Individuals who use 5G technology with robust safety measures, such as secure networks and protection against cybersecurity threats, are more likely to trust the technology and have confidence in its reliability^58^. People secure their personal information and adequately protect their data while using 5G technology with safety affordance; as a result, individuals are more likely to feel comfortable and willing to adopt it^54^. Safety affordance can also provide users with the necessary training and support to effectively and safely use 5G technology. This can involve educational programs, user manuals, or customer support services. When students feel adequately informed and supported, their ITU5G technology increases as they perceive a lower risk of encountering safety-related issues. Safety affordance can empower users by giving them a sense of control over the 5G technology^59^. When students believe they have control over the safety features and settings of 5G technology, such as the ability to customize privacy settings or limit data sharing, they are more likely to perceive it as safe and be motivated to use it^30^. A past study^60^ promoted the ITU5G technology among university students in China, it is essential for technology providers and stakeholders to prioritize safety affordance. Hence, we hypothesize that,

#### Hypothesis 3

Trustworthiness and ITU5G significantly mediate the relationship between safety affordance and AU5G.

### Moderating role of facilitation conditions

Facilitation conditions can create a favourable environment for telecommunication companies to invest in 5G infrastructure. It includes support from the government and other regulatory authorities, which is crucial for implementing the 5G technologies. Regulatory approval includes streamlining the licensing process, providing tax incentives, and reducing bureaucratic hurdles^4^. Supportive regulatory bodies facilitate investment in building and expanding 5G networks, increasing the availability and coverage. Facilitation conditions play a crucial role in allocating and managing spectrum, the radio frequencies used for wireless communication^61^. By ensuring sufficient and appropriate range is available for 5G deployment, regulators enable network operators to provide high-quality and reliable 5G services. When efficiently managed spectrum resources, it facilitates the deployment of 5G networks, leading to improved user experience and increased usage of 5G technology^62^.

5G technology brings new security and privacy challenges due to its increased complexity and connectivity^63^. The standards and guidelines are established due to favourable facilitation conditions that address these concerns and ensure that adequate security measures are adopted by network operators^21^. Regulatory support helps to create a competitive market for 5G services, leading to better pricing, service quality, and innovation. Effective supporting systems can facilitate implementation and prevent anti-competitive practices, such as monopolies or unfair market dominance, ensuring consumers have various choices and high-quality services. Users distinguish a competitive market with consumer protection measures in place; they are more likely to embrace 5G technology^64^.

Regulatory bodies can foster stakeholder collaboration, including industry players, academia, and standardization organizations^65^. By encouraging partnerships and cooperation, regulators promote the development of common standards and interoperability, which is vital for successfully implementing 5G technology. Individuals seeking information about 5G more actively may better understand its benefits and potential risks^66^. Regulatory bodies gather data about relevant concerns and collect information about 5G technology. Using 5G technology creates environmental awareness, such as energy consumption, electronic waste, and potential effects on ecosystems. This collaborative approach enhances the AU5G, ensuring seamless integration of devices, applications, and services across different networks and providers^67^.

Facilitation condition plays a crucial role in shaping the 5G ecosystem. By facilitating investment, ensuring spectrum availability, addressing security concerns, promoting fair competition, and fostering collaboration, regulators can positively influence the ITU5G technology among consumers and industry players. Regulatory authorities ensure safety affordance and the perceived level of safety and security that 5G technology provides to users^28^. Users considering 5G a safe and reliable technology are likelier to use it. Trust developed by government authorities as they provided a perception of reliability, credibility, and ethical practices associated with 5G technology. Trustworthy networks and service providers are more likely to gain users' trust with the help of regulatory support^68^.

Regulatory support can facilitate the deployment and expansion of 5G networks, ensuring broader coverage and accessibility in university campuses and surrounding areas. When students perceive that 5G is readily available and offers a seamless connectivity experience, they are more likely to embrace the technology and consider it for their day-to-day activities^1^. Regulations that mandate service providers to maintain a certain level of quality and performance in their 5G networks can enhance user experience. Students who experience faster speeds, lower latency, and better network reliability are more inclined to use 5G for bandwidth-intensive activities like streaming, online gaming, virtual reality, or accessing cloud-based resources, thereby increasing their ITU5G technology^41^.

Facilitation conditions foster an environment conducive to innovation and can encourage the development of new applications, services, and use cases that leverage the capabilities of 5G technology^69^. They can play a role in promoting fair pricing, competition, and affordability of 5G services. 5G plans and devices are reasonably priced and offer value for money; students are more likely to perceive them as a worthwhile investment and be more willing to adopt the 5G technology^70^. The previous studies stated that UTAUT2 models improve the explanatory power of the behavioural intention and use of 5G^25^. The organizational supportive facilitation conditions can strengthen the relationship between trustworthiness and ITU5G. So that universities with more facilitation conditions for 5G technologies can build stronger trustworthiness that enhances the students’ ITU5G. Therefore, we hypothesize that:

#### Hypothesis 4

Facilitation conditions strengthen the relationship between trustworthiness and ITU5G in university students.

**Figure 1:**
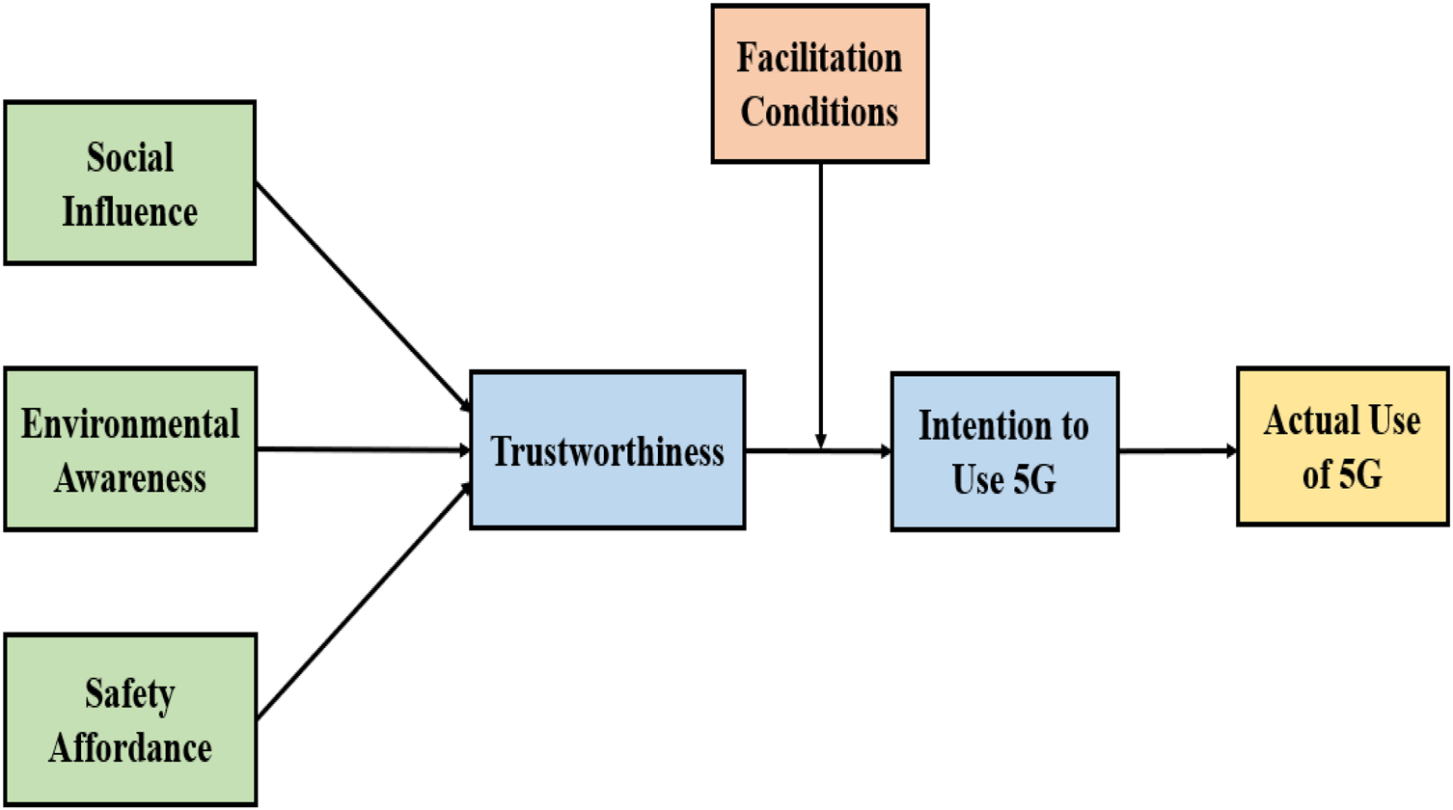
Conceptual framework.

Figure 1 explains the research model below, which depicts the literature review mentioned above.

## Measure and methods

The rapid advancement of technology has brought forth the emergence of 5G wireless network technology^71^, promising unprecedented speed, connectivity, and transformative potential across various sectors. 5G has gained significant attention in China, with its implementation and development being a national priority. As the usage of 5G technology becomes increasingly pervasive, it is essential to understand the factors influencing individuals' intention to use it^12^. The world's largest market for mobile communications is in China. In their major provinces, they are testing 5G. According to projections, 28% of mobile networks in China, or nearly one-third of all 5G connections worldwide, will use 5G technology by 2025^11^. As a result, Beijing will become the nation's leader in 5G usage. Therefore, an online poll was used to gather information from Chinese students from the top six universities in Beijing, including undergraduates, postgraduates, and researcher scholars. The study will use PLS-SEM to conduct a quantitative research design analysis of the correlations between the factors affecting the ITU5G. The survey will be available online in the last quarter of 2023 through various social media channels, email distribution lists, and personal relationships. We also collected the data from multiple sources, such as Wenjuanxing and Josump platforms. The researcher^25^ argues that an online survey for data collection provides a more rapid, efficient, and high-quality answer than an offline survey while reaching a target audience that would otherwise be challenging. 440 participants responded by sending in their submissions. 76 surveys were rejected because they were either incomplete or contained missing values. Finally, 364 (82%) responses were collected and used for analysis. According to Kline, this sample size is appropriate for SEM analysis^72^. Similarly, according to Roscoe's, a sample size greater than 30 and less than 500 is most suitable for behavioral and marketing research^73^. So, we have chosen a sample size within a range of 30–500 to get the authenticity of the current study. Further, the current study was assessed through a self-administered questionnaire that shows the relationship among variables. For this purpose, the questionnaire is divided into two sections: the first part represents the demographic information, which includes age, education, gender, locality, and specialization. All details of demographic profiles are given in Table 1.

**Table 1:** Demographic details.

Demographics	Distribution	n = 364
Gender	Male	206 (57%)
	Female	158 (43%)
Age	16–22 years	72 (20%)
	23–29 years	186 (51%)
	Above than 29 years	106 (29%)
Qualification	Bachelors	97 (27%)
	Maters	179 (49%)
	PhD	88 (24%)
Locality	Beijing	193 (53%)
	Shenzhen	71 (20%)
	Shanghai	66 (18%)
	Others	34 (9%)
Specializations	Management sciences	111 (31%)
	Business administration	124 (34%)
	Tourism management	129 (35%)

The second of the questionnaire is taken from previous research. To fit the context of our study, we changed the context of each item. A five-point Likert scale was used to evaluate each construct (strongly disagree = 1, strongly agree = 5). We conducted pilot research with 39 replies to confirm the item's validity and reliability. Further adjustments were made in light of the researchers' recognized reaction. Depending on the responders, all items were translated into English or Chinese. ITU5G, four items were occupied by^74^ and slightly modified. The five items of trustworthiness (TW) suggested by^18^. Four items about AU5G were taken from^23^. Safety affordance (SA) was calculated by five items taken from^7^ and used after minor modifications. The six items for social influence (SI) were collected from^10^. Environmental awareness (EA) of five items adapted from^5^. Facilitation conditions (FC) were assessed by four items taken from^12^. For data collection, a convenient sampling technique was used as an effective medium to get the appropriate results with ease of accessing the respondents^75^. Despite the less generalizability of results, the convenience sampling method is used due to easy access to the respondents and its relevance to the measurement item^76^. Hence, convenience sampling is not a major concern for our study. This study is free from common method bias as we have applied the CFA (confirmatory factor analysis) techniques to minimize the bias effect.

### Ethics statements and declarations

We confirm that all methods were carried out in accordance with relevant guidelines and regulations. Humans who participated in this study are aware of the purpose of the study, and their confidential information has not to be shared with anyone. All study participants provided their written informed consent. Study data is used after the consent of participants. The questionnaire used in this study started with the declaration and purpose of the study. Experimental protocol was approved by the ethical review board of the Beijing university of technology.

## Analytical framework and results

SEM was used for data analysis due to its component-centered nature; this method assessed all variables' relationships^77,78^. The researcher chose PLS-SEM for this inquiry because of its widespread use and applicability; later research^10^ offered evidence. Structural equation modelling (SEM) outperforms traditional statistical analysis methods. It improves statistical analysis's effectiveness, simplicity, and precision^79^. SEM is a multivariate analysis method that can help with the simultaneous evaluation of many variables concurrently. The SEM technique is becoming increasingly well-liked in business research because it can handle different interactions simultaneously^80^. A strong statistical method is required for the administration and social discipline investigation^81^. Inappropriate application of analytical techniques may lead to incorrect conclusions. Two phases of PLS-SEM analysis, such as measurement and structural models, have been considered by two stages of measurement findings^82^.

The measurement assessment model used in this study included estimations of the inner prototypical and consistency and rationality tests for different factors that influence people's ITU5G. The validity is determined by the item correlation by the Cronbach alpha, the composite reliability, and the item loading^76^. Nevertheless, discriminant validity pertains to the relationship between variables examined using cross-loading, Heterotrait-Monotrait ratio, and Fornell-Larcker^83^. The measurement model also incorporates the research analysis described in the section on outcomes and route analysis for testing hypotheses. The correlations between the components explored in this research have been identified by path analysis. The findings showed that social influence, environmental awareness and safety affordance positively impact AU5G through trustworthiness, supporting hypotheses H1a, H1b and H1c. Additionally, the results showed that facilitation conditions significantly moderate the relationship between trustworthiness and ITU5G in university students, which leads to AU5G accepting H2.

### Common method bias

In research methodologies based on surveys and including data gathered from a single source, common method variance (CMB) could be an important aspect to take into account^84^. The selection of the respondents was specifically based on their familiarity with the use of 5G. Because the survey questions were carefully designed and respondents answered them based on their opinions, it was less likely that respondents would provide thoughtless answers. Harman's one-factor test, as employed by^85^, was also used in this study to show that common method bias was absent. According to the results of the exploratory component analysis on all items for the variables, the single factor variance in our study was 33.4%, which is below the 50% criterion. This suggests that there was no evidence of a common method bias issue in the survey responses. Additionally, the^86^ technique—which postulates that a stronger correlation between the research variables would indicate the presence of common method bias—was also employed to demonstrate robustness in identifying common method bias. We used Smart-PLS in this study to measure inter-construct correlations, however there is no indication of common technique bias. Next, we examined the variance inflation factor (VIF) in this study using a modern method that was suggested by^87^. Every measured VIF value fell between less than five.

### Measurement assessment model

**Figure 2:**
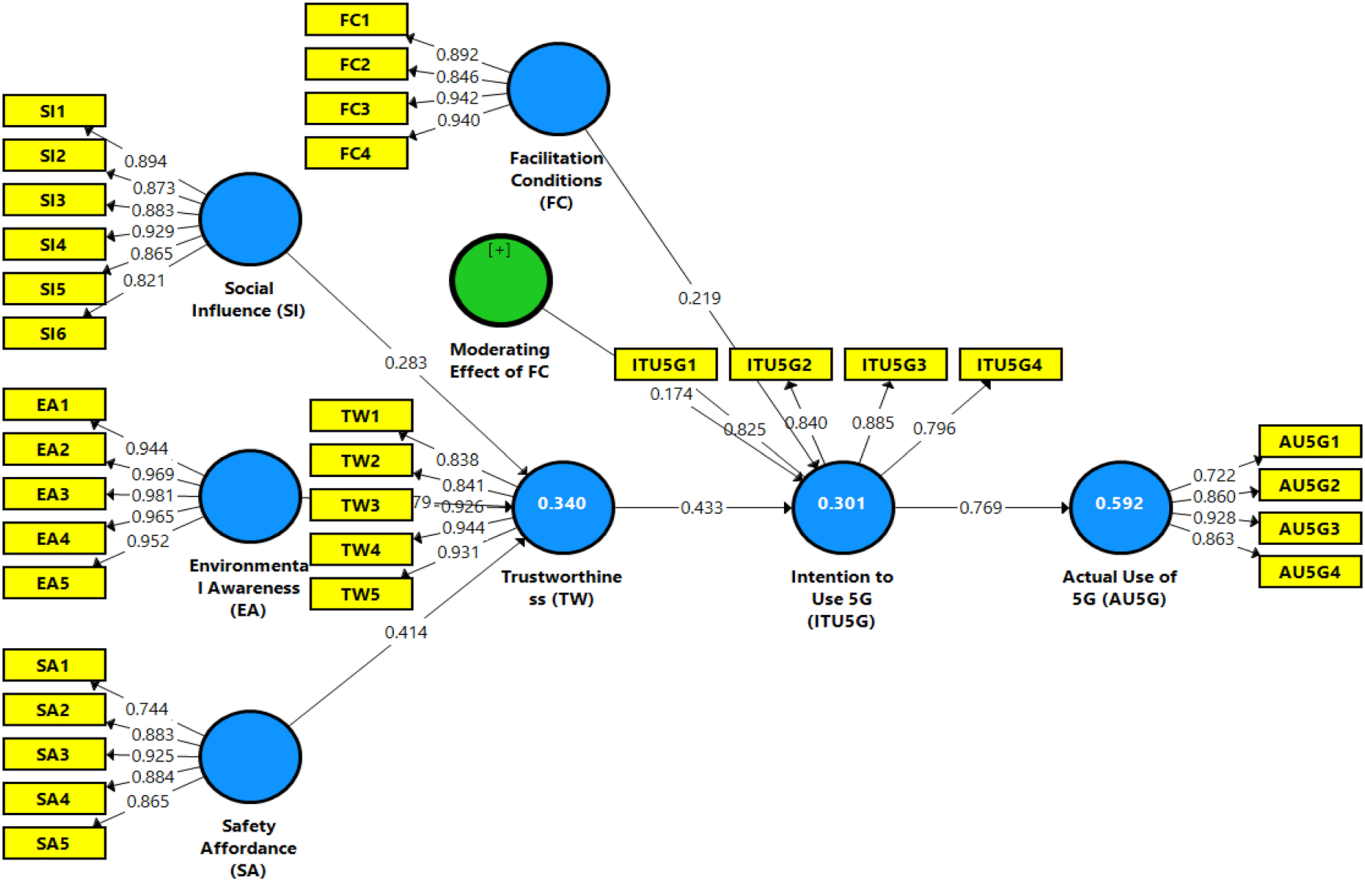
Measurement assessment model.

The measurement model examines the discriminant, reliability, and convergent validity among the defined constructs^80^. The measuring model validates the constructs' dependability and factor loadings and validates the constructs' validity^79^. The measurement assessment method is consistent across reliability and validity evaluations (convergent and discriminant validity). The average variance extracted (AVE), composite reliability (CR), Cronbach's alpha (α) and outer loading were used in this study to evaluate convergent validity, internal consistency reliability, and item reliability^68^. The item loadings are more than the criteria value of 0.7, as shown in Table 2. Every reflection was subsidized to the new variable, and the average factor loadings were more than 0.70^88^. AVE is higher than the suggested value of 0.5^89^. Each standard has an overall dependability rating greater than 0.70, which denotes accurate measurements. The results of the currently chosen 5G variables facilities show that all AVE values vary between 0.716 and 0.926. The CR values for all 5G constructs range between 0.909 and 0.984. The Cronbach's alpha values for all 5G constructs range between 0.865 and 0.980. While outer loadings of each construct range from 0.722 to 0.981. Tables 2 and 3 display this measurement model's validated reliability and validity values. Since all factor loading values are greater than 0.7, all items in the measurement assessment model have good convergent validity. The variable factor loadings and measurement assessment model are shown in Fig. 2.

**Table 2:** Factor loadings.

Construct	Item		FL	α	CR	AVE	Source
Intention to use 5G (ITU5G)				0.865	0.909	0.716	^74^
	ITU5G1	I am looking forward to the massive use of 5G networks	0.825				
	ITU5G2	I look forward to emerging 5G related products on the market	0.840				
	ITU5G3	I would like to learn how to use a 5G network	0.885				
	ITU5G4	I will try to use 5G related products	0.796				
Actual use of 5G (AU5G)				0.858	0.904	0.701	^23^
	AU5G1	I will continue to keep using 5G technology	0.722				
	AU5G2	I have used 5G technology which gives me benefits	0.860				
	AU5G3	As far as I know, 5G services are useful	0.928				
	AU5G4	I would recommend other people to use 5G	0.863				
Social influence (SI)				0.941	0.953	0.771	^10^
	SI1	I acquired information about 5G technology through websites, social media, and seminars	0.894				
	SI2	I gained information and understand the benefits in various domains	0.873				
	SI3	I feel understating the positive use of 5G reduction in perceived risks, such as privacy concerns	0.883				
	SI4	I assess more information of 5G are more likely to have the intention to use it in their daily lives	0.929				
	SI5	I get information about 5G is reliable from different social media platforms	0.865				
	SI6	I find the available information sources reliable, comprehensive, and accessible	0.821				
Environmental awareness (EA)				0.980	0.984	0.926	^5^
	EA1	I consider the potential environmental impact of my actions when making my decisions	0.944				
	EA2	I would like to describe myself as environmentally responsible	0.969				
	EA3	I am worried about wasting and destroying the earth's resources	0.981				
	EA4	Even if I feel inconvenient, I would like to take more environmentally friendly actions	0.965				
	EA5	I am very knowledgeable about 5G radiation and its related environmental issues	0.952				
Safety affordance (SA)				0.913	0.935	0.744	^7^
	SA1	I am anxious about 5G technology safety	0.744				
	SA2	I am concerned with the information about the 5G that I used	0.883				
	SA3	I frequently read about 5G technology	0.925				
	SA4	The superiority and safety of 5G these days alarms me	0.884				
	SA5	5G technology vendors can provide me with the knowledge to ensure good health	0.865				
Trustworthiness (TW)				0.939	0.954	0.805	^18^
	TW1	Based on my experience with the 5G services will have integrity	0.838				
	TW2	Based on my experience with the 5G services will be reliable	0.841				
	TW3	I trust that the 5G services will be displayed as expected	0.926				
	TW4	I trust that the 5G providers will provide enough safeguards to protect me from liability for damages	0.944				
	TW5	I believe that 5G vendors wish to be known for keeping promises	0.931				
Facilitation conditions (FC)				0.927	0.948	0.821	^12^
	FC1	I feel regulatory agencies keep my best interests in mind	0.892				
	FC2	I trust that the regulatory bodies will pass legislation for individual privacy concerns	0.846				
	FC3	I am confident that the government will protect my interaction with 5G services	0.942				
	FC4	I trust that the networking companies will improve services to serve me better	0.940				

FL: Factor loadings, VIF: Variance inflation factor, CR: Composite reliability, AVE: Average variance extracted, α: Cronbach's alpha.

**Table 3:** Discriminant validity.

Constructs	AU5G	EA	FC	ITU5G	SA	SI	TW
Fornell–Larcker criterion							
AU5G	**0.943**						
EA	0.105	**0.859**					
FC	0.557	0.126	**0.962**				
ITU5G	0.710	0.126	0.745	**0.830**			
SA	0.734	0.164	0.814	0.817	**0.849**		
SI	0.305	0.197	0.268	0.359	0.338	**0.875**	
TW	0.339	0.047	0.349	0.457	0.409	0.376	**0.899**
Heterotrait-monotrait ratio (HTMT)							
AU5G							
EA	0.110						
FC	0.576	0.136					
ITU5G	0.780	0.140	0.818				
SA	0.790	0.182	0.739	0.817			
SI	0.317	0.219	0.275	0.393	0.364		
TW	0.355	0.047	0.365	0.511	0.441	0.394	

EA: Environmental awareness, SI: Social influence, ITU5G: Intention to use 5G, FC: Facilitation conditions, SA: Safety affordance, TW: Trustworthiness, AU5G: Actual use of 5G.

Square root of AVE is displayed on the diagonal and bold values.

The study research^90^ confirmed that the benchmark of the values of discriminant validity is less than 0.85 or 0.90, that reflect complete standards are less than 0.90. This study has also shown that the link between components has discriminant validity. The outcomes of this study also analyze the relationship between variables deemed to have discriminant validity. Initially, the Fornell-Larcker and cross-loading procedures were used to assess the discriminant validity in Table 3. These data indicated a weak correlation between variables and supported the selection of all 5G as discriminant valid. Table 3's bold values show that overall influences have strong links with some other components but substantial correlations with others^87^. The bold values of the cross-loadings table were contrasted with other factors in each column for the test discriminant validity^24^. This study analysis shows strong discriminant validity and is more significant than row-wise other components. Second, the HTMT ratio and other cutting-edge techniques have been used to test discriminant validity. The data shows that the HTMT ratio is less than 0.85^79^. These findings in Table 3 indicate a valid relationship between factors and discriminant validity.

### Structural assessment model

The measurement model is the initial step of the SmartPLS and is followed by the structural assessment model. The second stage was conducted in this instance, examining the relationship between exogenous and endogenous variables. Numerous statistical data, including effect size (f^2^), t values, predictive relevance (Q^2^), coefficient of determination (R^2^), and path coefficient (values), are presented by the structural assessment model^90^. Similarly, the variance inflation factor (VIF) is evaluated for more appropriate results, which displays the multicollinearity among constructs. The acceptable range of multicollinearity is less than 5, which shows that all the variables in the study are highly correlated. Criteria for rating hypotheses and estimating the significance of path coefficients are provided in the PLS-SEM literature. 5000 subsamples were used in a bootstrapping method with a 5% significance level (one-tailed) to assess the importance of the hypotheses. Similarly, t values greater than 1.64 indicate a significant relationship between the variables in the model^83^. The values of all variables are positive indicators in the structural assessment model for AU5G and have a significant association*.*

On the other hand, according to^83^, the R^2^ value is not a suitable and efficient technique for assisting a model. The analytical significance dimension Q^2^ of the prototype is thus an applicable technique. The dormant exogenous values with disproportionate prognostic importance^7^ show that the value of Q^2^ is more complex than zero. The Q^2^ result of 0.522 demonstrates the model's high level of predictive power and the fact that it is increasing the 5G facilities among individuals. According to^91^, typical values for f^2^ are 0.061, 0.204, and 1.450, which denote mild, medium, and considerable impacts. The value of f^2^ was calculated assuming that the effect size ranges from moderate to large.

Moreover, the standardized root mean square residual (SRMR) is the difference between the studied interrelationship and the model indirect interrelationship matrix. An excellent match in SRMR is indicated by values less than 0.10 or 0.08. SmartPLS is used to generate the structural model, and model fitness is assessed. The fitness indices' findings often indicate that the model fits well. The values listed below serve as an example: SRMR = 0.061, Chi-square = 2311.134, and NFI = 0.817. A list of several statistical techniques is provided in Table 4.

**Table 4:** Model fit.

	Saturated Model	Estimated Model
SRMR	0.061	0.135
d_ULS	2.059	10.163
d_G	1.352	1.675
Chi-Square	2311.134	2620.447
NFI	0.817	0.792

The hypothesis testing outcomes showed that trustworthiness and ITU5G positively mediate the relationship between social influence and AU5G with values (β = 0.094, t = 4.673, and *p* < 0.05). In the same way, trustworthiness and ITU5G positively mediate the relationship between environmental awareness and AU5G with values (β = 0.060, t = 3.705, and *p* < 0.05). Similarly going forward, trustworthiness and ITU5G positively mediate the relationship between safety affordance and AU5G with values (β = 0.138, t = 4.946, and *p* < 0.05). Therefore, H1a, H1b and H1c are accepted. For moderation results, findings reveal that facilitation conditions significantly moderate the relationship between trustworthiness and ITU5G in university students (β = 0.174, t = 3.543, and *p* < 0.05). Thus, H2 is accepted. The R^2^ value for TW, ITU5G, and AU5G is 0.340, 0.301, and 0.592, showing that the model can considerably enhance use of 5G as output in Table 5.

**Table 5:** Hypothesis testing.

Hypothesis and relationship	β-values	STDEV	t-values	*p*-values	Decisions
Panel A. Main effects					
SI → TW	0.283	0.054	5.258	0.000	Supported
EA → TW	0.179	0.051	3.503	0.001	Supported
SA → TW	0.414	0.047	8.753	0.000	Supported
TW → ITU5G	0.433	0.046	9.366	0.000	Supported
ITU5G → AU5G	0.769	0.029	26.834	0.000	Supported
FC → ITU5G	0.219	0.045	4.825	0.000	Supported
Panel B. Mediating effects					
SI → TW → ITU5G → AU5G (H_1a_)	0.094	0.020	4.673	0.000	Supported
EA → TW → ITU5G → AU5G (H_1b_)	0.060	0.016	3.705	0.000	Supported
SA → TW → ITU5G → AU5G (H_1c_)	0.138	0.028	4.946	0.000	Supported
Panel C. Moderating effects					
TW*FC → ITU5G (H_2_)	0.174	0.049	3.543	0.000	Supported
Panel D. R^2^					
TW R^2^ = 0.340					
ITU5G R^2^ = 0.301					
AU5G R^2^ = 0.592					

EA: Environmental awareness, SI: Social influence, ITU5G: Intention to use 5G, FC: Facilitation conditions, SA: Safety affordance, TW: Trustworthiness, AU5G: Actual use of 5G.

## Discussion and recommendations

This study identifies statistically important social, environmental, psychological, and intrinsic elements that influence the intention of individuals to use 5G technology. Understanding consumer intention and behavior in technology adoption might start with these elements. The five main factors of 5G adoption are information acquisition, environmental awareness, safety affordance, trustworthiness, and regulatory support, which contribute to catching people's ITU5G. This study suggests that these components can be improved if carefully developed and significantly impact adopting 5G in Chinese university students. To increase sales or the rate of 5G adoption, stakeholders must direct their attention appropriately. This study examined the relationship between social influence, environmental awareness and safety affordance through trustworthiness and ITU5G and ultimately challenged the AU5G. Additionally, this study examined the moderating role of facilitation conditions to measure the strength of ITU5G when a trustworthy relationship is developed among the users (Chinese students). 364 participants of university students empirically test the foundations of the concept. The investigation came to four important conclusions.

Firstly*,* this research study has inspected the mediating role of trustworthiness and ITU5G between social influence and AU5G. The findings proved that social influence is positively associated with the AU5G, supported by many past studies^9,10^, but its mediating role in terms of trustworthiness and ITU5G is not yet been explored in any prior studies^39,40^. However, the results suggested that social influence such as peer feedback, student reviews of technology usage, online communities and social media platforms are the main sources that help build trust among Chinese students regarding the intention to adopt the 5G technology. A past study^15^ highlighted individuals have a positive attitude toward using 5G and express a desire to adopt it; they are more likely to actively seek out information about its features, benefits, and implementation. Individuals are aware of China's advancements in 5G and the associated benefits; it can create a sense of national pride and a desire to be part of the technological progress, thus positively influencing their ITU5G. A prior study^5^ argued that social influence helps individuals become aware of the capabilities, benefits, and potential applications of 5G technology. People are more likely to adopt a positive attitude toward utilizing 5G if they know its benefits, which include faster data rates, reduced latency, and support for cutting-edge technology like the Internet of Things and driverless vehicles^34^. Recent research^37^ also focused on the trustworthy factor of 5G technology usage, which ultimately improves communication, enhanced productivity, better access to services, and transformative applications in various sectors like healthcare, transportation, and entertainment. China has been actively promoting the development and adoption of 5G technology. Through government initiatives and facilities, there is a strong focus on creating an ecosystem that supports 5G infrastructure and services^92^.

Secondly, this research study has inspected the mediating role of trustworthiness and ITU5G between environmental awareness and AU5G. The findings proved that environmental awareness is positively associated with trust to use the technology, supported by many past studies^46,47^, while its mediating role in terms of trustworthiness and ITU5G is not discussed yet in the above context^7^. This finding implies that individuals concerned about the environment may view 5G technology as a means to promote sustainability or reduce environmental impact. A prior study^70^ focused on the potential benefits of 5G, such as its ability to enable smart city initiatives, improve energy efficiency, and enhance environmental monitoring and management. 5G technology is a critical enabler of various smart solutions, such as smart grids, smart transportation systems, and smart buildings^93^. These solutions promote sustainability by optimizing resource utilization, reducing emissions, and improving efficiency. Similarly, another researcher^5^ expressed that as 5G technology can enable these eco-friendly technologies, environmentalists may be more likely to adopt them. The pandemic has highlighted the importance of remote connectivity with 5G technology; people can access high-speed internet and perform bandwidth-intensive tasks from remote locations. This capability reduces the need for commuting and physical travel, contributing to decreased carbon emissions^48^. Real-time data collection, analysis, and transmission are made possible by 5G technology, improving environmental monitoring systems, tracking wildlife, and natural resource management. Environmentally conscious people may appreciate the usefulness of 5G technology in advancing environmental conservation initiatives^6^.

Thirdly, the current study has examined the mediating role of trustworthiness and ITU5G between safety affordance and AU5G. The findings proved that safety affordance is positively associated with the AU5G, supported by many past studies^7,36^, and its direct association with trust in terms of 4G technology usage is also discussed in earlier research^7,23^. Although safety affordance is measured in multiple contexts in technology literature^54^ its role in the context of 5G technology actual usage is under-researched. In a past study^70^, the term safety affordance described the capabilities and attributes of a given technology that are considered safe. Security precautions, data security, privacy restrictions, and general technology reliability are some of these qualities. Safety affordance positively influences ITU5G, showing that people are more likely to have a good attitude toward and express an intention to utilize 5G technology when they believe it to be safe and secure^94^. This finding aligns with the general understanding that perceived risks and concerns associated with new technologies can be significant barriers to adoption. According to earlier research^37^ by providing users with safety features like strong security safeguards and open privacy rules, technology providers can increase their trust and confidence, increasing their desire to use the technology. Safety affordances that address these concerns, such as adhering to established safety guidelines and regulations, conducting thorough risk assessments, and transparently communicating the safety measures taken, can help alleviate fears and increase users' confidence in using 5G^95^. Safety features that allow consumers to manage their exposure to 5G radiation, such as changeable power levels or signal strength information, can improve user impression of safety. Users' willingness to adopt and use 5G technology may rise if they feel in control of their exposure and can adjust their settings to their comfort level^63^.

Fourthly, this research study has examined the moderating association of facilitation conditions between trustworthiness and ITU5G. The findings proved that facilitation conditions are positively moderated between trustworthiness and ITU5G argument supported by multiple studies^12,95^. A past study^96^ discussed that information acquisition in the context of 5G technology refers to learning about and comprehending its characteristics, advantages, and potential drawbacks. Users can gain trustworthy information with the support of clear guidelines, regulations, and communication from regulatory organizations, which may favourably impact their decision to use 5G^53^. Users should consider environmental issues, including the effect on humans, electromagnetic radiation, or energy usage, when deploying and using 5G networks. Establishing standards and guidelines for environmentally sustainable 5G infrastructure can alleviate these worries. In Chinese universities familiar with technological breakthroughs, it is difficult to highlight the conditions that moderate the trustworthiness-ITU5G relationship is crucial. A university with vigorous facilitation conditions, such as innovative infrastructure and educational programs focused on 5G, may increase the trustworthiness and acceptance of the technology among its students. A prior study^7^ established that safety affordance describes a technology's perceived capacity to offer a secure and safe user experience. By defining standards and regulations for network infrastructure, data protection, and privacy, regulatory assistance plays a crucial role in assuring the security and safety of 5G networks^55^. Users' level of trust in the 5G ecosystem's dependability, security, and integrity is their level of trustworthiness^68^. Users' faith in 5G technology increases when they believe the regulatory framework is solid and reliable, positively impacting their desire to use it.

### Theoretical and practical implications

The theoretical and practical implications provide valuable insights for policymakers, communication strategists, and technology providers seeking to promote the acceptance and adoption of 5G technologies. Regarding theoretical implications, this research emphasizes the importance of facilitation conditions as a moderating variable in the context of 5G adoption. By building confidence, addressing safety issues, and guaranteeing environmental sustainability, regulatory organizations help to facilitate the acceptance and use of new technologies. The fact that environmental consciousness is now considered a factor in 5G intention demonstrates the growing significance of sustainability in technological adoption. This study highlights the importance of considering ecological considerations when developing technological acceptance models and any potential environmental advantages or dangers of 5G. Reliability and safety affordance are important variables influencing the intention to utilize 5G. This study adds to our understanding of how trust develops in the context of new technologies by emphasizing the need for a dependable and secure 5G infrastructure to promote user acceptance. This study contributes to expanding the UTAUT2 paradigm by including social influence, environmental awareness, safety affordance and trustworthiness as factors of intention to utilize 5G. It deepens our comprehension of the intricate forces influencing people's attitudes and behavioural intentions toward developing technologies.

This study has some practical implications. The findings highlight the need for regulatory organizations to offer assistance and direction to guarantee a seamless and effective deployment of 5G. Policymakers can use the results of this study to create rules that handle safety issues, ensure environmental sustainability, and promote user trust, all of which will help 5G technology become more widely used. Effective communication strategies should concentrate on giving precise and easily accessible information about the technology's advantages, safety precautions, and environmental impact to increase people's intention to adopt 5G. Stakeholders, such as tech companies and regulatory bodies, can work together to communicate accurate information and address any misunderstandings or worries about 5G. This study emphasizes the value of putting money into a reliable and secure 5G infrastructure to win over potential users. Prioritizing safety and security procedures can help service providers deliver the benefits of technology while limiting risks and vulnerabilities. The study emphasizes the importance of considering environmental elements while developing and deploying 5G networks. Collaboration between technology providers and authorities is necessary to implement eco-friendly procedures and spread knowledge about the potential benefits of 5G for sustainability. Regulators and policymakers need to understand how important it is for them to support the introduction of 5G technology. The study examined the need to encourage regulatory regimes that balance environmental concerns, consumer protection, and innovation. These frameworks should support ethical business practices, safeguard personal information, and set up systems for monitoring and enforcing compliance. Additionally, exploring the factors that may hinder or facilitate the adoption and use of 5G technologies in real-world settings would contribute to developing targeted interventions and strategies to promote its acceptance and usage.

### Conclusion and policy recommendations

This study investigated the impact of social influence, environmental awareness, and safety affordance on actual use of 5G among Chinese university students through trustworthiness and intention to use 5G and the moderating role of facilitation conditions. This research shed light on the factors influencing the acceptance and adoption of 5G technology in the Chinese context. The results revealed four significant findings. Firstly, trustworthiness and ITU5G positively mediate the relationship between social influence and AU5G. This study suggests that when Chinese university students get positive feedback from social media platforms and peers about adopting 5G technology, they are likelier to develop a positive attitude towards its actual usage. Secondly, trustworthiness and ITU5G positively mediate the relationship between environmental awareness and AU5G. Environmental awareness about technology usage develops trust among Chinese university students to adopt and utilize the 5G. It is derived from the current study that Chinese university students who understand the potential environmental benefits of 5G technology are more willing to perceive it as advantageous and express a stronger intention to use it. Thirdly, trustworthiness and ITU5G positively mediate the relationship between safety affordance and AU5G. This suggests that when Chinese university students perceive 5G technologies as safe and secure, they feel trustworthy and reliable and are more inclined to adopt AU5G. Fourthly, facilitation conditions were found to significantly moderate the relationships between trustworthiness and ITU5G in shaping the AU5G among Chinese university students. This implies that the influence of information acquisition, environmental awareness, safety affordance, and trustworthiness on the intention to and real-time usage of 5G technology is contingent upon the level of facilitating conditions provided by the government and regulatory authorities. When government facilities are satisfactory, the positive effects of these factors on the ITU5G are strengthened. By considering these factors and providing facilitating conditions, policymakers and stakeholders can foster a favourable environment for the adoption and usage of 5G technologies among university students in China.

### Limitations and future directions of this study

This study has some limitations and future directions. Firstly, this study focused solely on Chinese university students, which may limit the generalizability of the findings to other populations, such as non-students or individuals from different cultural backgrounds. Future studies should consider including a more diverse sample to enhance the external validity of the findings. Secondly, this study employed a cross-sectional design, which captured data at a single point in time. This limits the ability to establish causal relationships between the variables. Future research could adopt a longitudinal design to examine the changes in ITU5G technologies over time and better understand the variables' causal relationships. Thirdly, this study relied on self-reported measures subject to response biases such as social desirability and memory recall. Future studies could incorporate objective measures or observational data to enhance the reliability and validity of the findings. Fourthly, we have collected data and conducted a comparative analysis of Chinese university students' ITU5G. Future studies would provide valuable insights for policymakers and practitioners in tailoring strategies to promote AU5G across different cultural contexts or countries. Fifthly, in this study, we have used moderators such as facilitating conditions that could influence the relationships between the variables. Future studies may focus on additional moderators, such as regulatory environment technological literacy. Lastly, we used a questionnaire to inspect all variables in this study. Future research employing mixed-methods approaches, combining qualitative and quantitative data, could provide a richer and more nuanced understanding of the factors influencing the intention to use.

## Data Availability

The raw data supporting the conclusions of this article will be made available by the authors, without undue reservation. Data can be obtained upon reasonable request by Co-author, Muhammad Farrukh Shahzad (farrukhshahzad207@gmail.com).
