# Author Correction: Effect of social influence, environmental awareness, and safety affordance on actual use of 5G technologies among Chinese students

**DOI:** 10.1038/s41598-024-58938-3

**Published:** 2024-04-11

**Authors:** Muhammad Farrukh Shahzad, Shuo Xu, Kanwal Iqbal Khan, Muhammad Faisal Hasnain

**Affiliations:** 1 College of Economics and Management, Beijing University of Technology, Beijing, 100124 People’s Republic of China; 2 Department of Management Sciences, University of Engineering and Technology, New Campus, Kala Shah Kaku, Pakistan; 3 Department of Chemistry, University of Agriculture, Faisalabad, 38000 Pakistan

Correction to: *Scientific Reports* 10.1038/s41598-023-50078-4, published online 17 December 2023

The original version of this Article contained errors in Table 2 and in the Reference list.

In Table 2, for the construct “Actual use of 5G”, the ‘Cronbach's alpha’, ‘Composite reliability’, and ‘Average variance extracted’ values were omitted. Additionally, the source was incorrect. The correct and incorrect values appear below.

Incorrect:

**Table:** 

Construct	Item		FL	α	CR	AVE	Source
Actual use of 5G (AU5G)							[26]
	AU5G1	I will continue to keep using 5G technology	0.722				
	AU5G2	I have used 5G technology which gives me benefits	0.860				
	AU5G3	As far as I know, 5G services are useful	0.928				
	AU5G4	I would recommend other people to use 5G	0.863				

Correct:

**Table:** 

Construct	Item		FL	α	CR	AVE	Source
Actual Use of 5G (AU5G)				0.858	0.904	0.701	[23]
	AU5G1	I will continue to keep using 5G technology	0.722				
	AU5G2	I have used 5G technology which gives me benefits	0.860				
	AU5G3	As far as I know, 5G services are useful	0.928				
	AU5G4	I would recommend other people to use 5G	0.863				

Due to an error during manuscript preparation, all References were interchanged. In addition, the Authors omitted the below Reference, which is now listed as Reference 2:

F. Rizzato, 5G users on average consume up to 2.7× more mobile data compared to 4G users (2020).

The previous Reference 2 is retained as Reference 7:

Shah, S. K., Zhongjun, P. T., Oláh, J., Popp, J. & Acevedo-Duque, Á. The relationship between 5G technology affordances, consumption values, trust and intentions: An exploration using the TCV and S-O-R paradigm. *Heliyon* 10.1016/j.heliyon.2023.e14101 (2023).

As a result, all subsequent references have been renumbered and the Reference list has been reorganized to ensure all references are cited at the correct position in the text.

The original Article has been corrected.

